# Responsiveness and Predictive Ability of the Chinese Version of the Action Research Arm Test in People with Cerebral Infarction

**DOI:** 10.1155/2019/8270187

**Published:** 2019-10-07

**Authors:** Jiang-Li Zhao, Tao Zhang, Zhi-Qin Xu, Ming-Hui Ding, Yan Leng, Rui-Hao Bian, Yu-Rong Mao, Dong-Feng Huang

**Affiliations:** ^1^Department of Rehabilitation Medicine, The First Affiliated Hospital, Sun Yat-sen University, Guangzhou 510080, Guangdong Province, China; ^2^Department of Rehabilitation Medicine, The Seventh Affiliated Hospital, Sun Yat-sen University, Shenzhen 518107, Guangdong Province, China

## Abstract

**Purpose:**

To detect the responsiveness and predictive ability of the Chinese version Action Research Arm Test (C-ARAT) in participants within the first 3 months after cerebral infarction.

**Methods:**

Ninety-seven individuals (75 men, mean age 59.87 ± 10.94 years) with a first cerebral infarction were enrolled in this study. The participants were evaluated by two outcome measures: C-ARAT and the Barthel Activities of Daily Living Index (BI) at five time points: 0D, 3W, 3M, 6M and 1Y after enrolment. The standardised response mean (SRM) and the Wilcoxon signed rank test were used to analyse responsiveness. Predictive validity was determined by using Spearman's rank correlation coefficients. The predicted performance of C-ARAT on activities of daily living (ADLs) was measured by linear regression model. Floor and ceiling effects were estimated by counting the proportion of subjects falling outside the 5% lower or upper boundary, respectively.

**Results:**

The C-ARAT showed moderate to large responsiveness in detecting changes over time (SRM = 0.58–0.84). The C-ARAT subscales showed small to large responsiveness (SRM = 0.44–0.90). The C-ARAT at 0D showed moderate to good correlation with the BI scores at 3W, 3M and 6M (*ρ* = 0.561–0.624, *p* < 0.001), and exhibited fair correlation with the BI score 1Y after enrolment (*ρ* = 0.384, *p* < 0.05). C-ARAT was a good predictor (adjusted *R*^2^ = 0.185–0.249) of BI within 3M follow-up. The C-ARAT total score showed a notable floor effect at 0D and 3W and a notable ceiling effect at 3M, 6M and 1Y.

**Conclusion:**

The results of this study support the use of the C-ARAT as a measurement of upper extremity function in individuals with a first cerebral infarction.

## 1. Introduction

Stroke is a leading cause of adult disability across the globe [1]. Stroke survivors are often left with severe upper extremity (UE) impairments and become dependent on others for activities of daily living (ADLs) [2]. One study found that the impairment of upper limb function in stroke survivors was the greatest barrier to independent daily living and return to the community [3]. An assessment tool with excellent responsiveness could aid in measuring the recovery progress of individual patients. There has been an increasing emphasis on the importance of investigating the responsiveness of a measurement tool [4–7]. In addition, the floor and ceiling effects reflect the extent to which scores cluster at the bottom and top of the scale range [5], and to some extent, indicate the best applicable population of the scale. Moreover, an optimal predictor would enable investigators to make sound prognostic decisions and facilitate planning for patient placement after discharge [8].

Several measures are used in the clinic for assessing UE impairment or disability [9–13]. One of the most commonly used measures for stroke survivors, the Action Research Arm Test (ARAT) [14], is implemented to evaluate UE performance, especially the fine motor function of the hand. To meet the clinical demand in China, we translated the original ARAT into a Chinese-version ARAT (C-ARAT) and examined the concurrent validity and reliability of the C-ARAT [15, 16]. However, no study has yet been directed towards detecting the responsiveness or predictive ability of the C-ARAT.

To further examine the psychometric properties of the C-ARAT, the aim of this study was to detect the responsiveness, predictive ability for ADLs and floor and ceiling effects of the C-ARAT in people with early cerebral infarction.

## 2. Methods

### 2.1. Translation

The original ARAT and its manual were translated from English to Chinese using a forward-backward procedure by an expert group. The translation protocol was published in a previous article [15].

### 2.2. Subjects

The subjects for this study, recruited by a convenience sampling method, were inpatients in the Department of Rehabilitation Medicine, the First Affiliated Hospital of Sun Yat-sen University, China, from August 2014 to December 2018. The inclusion criteria were as follows: (1) the occurrence of a first cerebral infarction with unilateral hemiparetic lesions confirmed by magnetic resonance imaging or computed tomography; (2) an interval of <3 months after cerebral infarction; (3) age of 40–80 years; (4) ability to maintain a sitting position for >30 min; (5) no severe deficits in communication, memory and understanding; (6) no additional medical, cardiovascular or orthopaedic condition or significant UE peripheral neuropathy; (7) willingness to participate in this study and sign the informed consent.

The participants' demographic details and major comorbidity data were collected from medical records.

This study was approved by the Human Subjects Ethics Subcommittee of the First Affiliated Hospital, Sun Yat-sen University, China. Informed written consent was obtained from all of the participants.

### 2.3. Procedure

Prior to baseline data collection, an experienced physiotherapist with 9 years of clinical experience in stroke rehabilitation was trained to properly administer the C-ARAT and the Barthel ADLs Index (BI). The C-ARAT and BI were administered at five time points: the first day of hospitalisation and enrolment (0D), 3 weeks after enrolment (3W), 3 months after enrolment (3M), 6 months after enrolment (6M) and 1 year after enrolment (1Y). The scores of the C-ARAT measured at 0D, 3W, 3M, 6M and 1Y were used to analyse the responsiveness and floor and ceiling effects. The BI scores at 3W, 3M, 6M and 1Y were used as the criteria for examining the predictive ability of the C-ARAT measured at 0D. The outcome measures were conducted in a random order by lucky draw in a quiet room. During the evaluation, participants could take sufficient rest to avoid the influence of fatigue caused by the assessment. Thus, the entire assessment took approximately 20–30 minutes.

### 2.4. Outcome Measures

#### 2.4.1. C-ARAT

The ARAT is a performance test for evaluating arm and hand function and dexterity after stroke [11]. Many studies have proven that the ARAT has good psychometric properties [5, 7, 17–24], showing valid (*ρ* = 0.73–0.97) [7, 20, 23, 24] and reliable (inter-rater reliability = 0.92–0.99, intra-rater reliability = 0.97–0.99) [18–21] efficacy in people with stroke. Yozbatiran et al. [18] presented a standardised approach along with a detailed test manual, which was translated by our expert group into a Chinese version according to a standard forward-backward translation protocol [15]. The C-ARAT includes 19 items categorised into four subscales: grasp subscale (items 1–6), grip subscale (items 7–10), pinch subscale (items 11–16) and gross-movement subscale (items 17–19). Each item is graded on a four-point original scale as follows: 0, unable to complete any part of the task within 60 s; 1, partial performance of the task within 60 s; 2, completion of the task but with great difficulty or in an abnormally long time (5–60 seconds) or 3, normal performance of the task within 5 seconds [18]. UE function is assessed unilaterally, beginning with the unaffected upper extremity.

#### 2.4.2. The Barthel ADLs Index (BI)

The BI is a measure of ability in basic ADLs [25]. The reliability, validity and responsiveness of the BI in subjects with stroke are well established (validity, *ρ* ≥ 0.92; inter- and intra-reliability, intra-class correlation coefficient ≥0.83) [26, 27]. Previous studies found that the BI and the Functional Independence Measure (FIM) showing similar psychometric characteristics for patients with multiple sclerosis or stroke or in patients undergoing rehabilitation [28, 29]. Furthermore, the BI is quicker and simpler to rate than the FIM. The BI thus seems to be preferable to the FIM motor subscale in measuring basic ADL after stroke [26]. It was used as an external criterion to calculate the predictive ability of the C-ARAT in this study. It comprises 10 items with a total score ranging from 0 to 100.

### 2.5. Statistical Analysis

All of the statistical analyses were performed using SPSS version 20.0. All of the tests applied were two-tailed. The level of significance was set at a *p* value <0.05.

#### 2.5.1. Participants

Demographic and clinical characteristics of participants of this study (*n* = 97) were demonstrated by descriptive statistics. The distribution of all the data was subjected to the Shapiro-Wilk test.

#### 2.5.2. Floor and Ceiling Effects

Floor and ceiling effects are defined as the mean percentages of subjects who scored beyond the lower and upper boundaries of the total score. The cutoffs for the floor and ceiling effects were set at 5% of the total score [21]. Therefore, scores <3, <1, <1, <1, and <1 points in the C-ARAT, grasp, grip, pinch and gross movement, respectively, were determined as a floor effect. Scores >54, >17, >11, >17 and >8 points on the C-ARAT, grasp, grip, pinch and gross movement were determined as a ceiling effect. Floor or ceiling effect >20% of the sample size was considered significant [20].

#### 2.5.3. Responsiveness

Responsiveness is defined as the ability to detect clinical differences [30, 31]. In this study, to evaluate the ability of the C-ARAT to detect changes in motor function, two approaches were used to examine the responsiveness during four sessions: 0D–3W, 0D–3M, 0D–6M and 0D–1Y. First, the standardised response mean (SRM), a type of effect size, is defined as the mean change in score divided by the standard deviation of the changed scores [32]. According to Cohen criteria [23, 33], an SRM ≥ 0.8 is large, 0.5 ≤ SRM < 0.8 is moderate and 0.2 ≤ SRM < 0.5 is small. Second, to determine the significance of the change in a more conservative way, the Wilcoxon matched-pairs signed-rank test was performed [7].

#### 2.5.4. Predictive Ability

Predictive validity was used to detect whether the total and each subscale in the C-ARAT was significantly correlated with certain future criterion measures [8]. Pearson or Spearman rank correlation coefficients (*ρ*) were used to calculate the correlation between C-ARAT on 0D and BI at all the follow-up time points as appropriate. The *ρ* values between 0 and 0.25 were considered low; *ρ* values between 0.25 and 0.50 were considered fair; *ρ* values between 0.50 and 0.75 were considered moderate to good; and *ρ* values >0.75 were considered good to excellent correlations [8]. If there was a significant correlation between C-ARAT and BI, the linear regression model with the “enter” method was performed to examine what proportion of the variability in BI scores at 3W, 3M, 6M and 1Y could be explained by the C-ARAT at enrolment [34].

## 3. Results

### 3.1. Demographics

Ninety-seven individuals with a first cerebral infarction were enrolled in this study. Of the 97 subjects who met the inclusion criteria and began to participate in the study, 6 individuals were lost to follow-up at 3 weeks after enrolment. There were 38 individuals who could not return to the hospital because of transportation difficulties at 3 months, 55 at 6 months and 62 at 1 year after enrolment. Thirty-five participants completed all of the assessments.  Table 1 details the demographic and clinical characteristics of participants at five time points. The Shapiro-Wilk test showed that the data were not normally distributed in this study.  Table 1 also gives the details of the demographic and clinical characteristics of the subjects who were lost to follow-up and the subjects who completed all of the assessments. The results show no significant difference in age at onset, sex, affected side, C-ARAT (including total, grasp, grip, pinch and gross movement) and BI between those two groups (*p* > 0.05).

### 3.2. Floor and Ceiling Effects

Table 2 shows the detailed results of the floor and ceiling effects analysed at five time points. The C-ARAT total score and gross-movement subscale score showed a notable floor effect at the first two time points (0D and 3W), and showed a notable ceiling effect at the latter three time points (3M, 6M and 1Y). The grasp, grip, pinch subscales showed notable floor effects at all five time points (0D, 3W, 3M, 6M and 1Y), and showed notable ceiling effects at the latter two time points (6M and 1Y), but the grasp subscale also showed a notable ceiling effect at the 3M time point.

### 3.3. Responsiveness

Table 3 shows the detailed results of the responsiveness analyses of the four sessions. The C-ARAT had moderate responsiveness in detecting changes in the first two sessions (0D–3W, SRM = 0.58; and 0D–3M, SRM = 0.72), and had large responsiveness in the latter two sessions (0D–6M, SRM = 0.81; and 0D–1Y, SRM = 0.84). Among the four subscales, the pinch subscale had the lowest responsiveness at each session, with the effect sizes from 0.44 to 0.65, indicating small to moderate responsiveness; the gross-movement subscale had the highest responsiveness during the first three sessions (0D–3W, 0D–3M and 0D–6M), with the effect sizes from 0.58 to 0.82, indicating moderate to large responsiveness; the grip subscale, similar to the total score, also had moderate responsiveness during the first two sessions (0D–3W, SRM = 0.53; and 0D–3M, SRM = 0.73), and had large responsiveness during the latter two sessions (0D to 6M, SRM = 0.80; and 0D–1Y, SRM = 0.90). The results suggest that the C-ARAT was able to detect small changes in subjects with a first-onset cerebral infarction.

### 3.4. Predictive Ability

Table 4 shows the detailed results of the predictive analyses. The C-ARAT total and subscales scores at 0D had moderate to good correlation with the BI score at 3W, 3M and 6M with *ρ* value from 0.521 to 0.624 (*p* < 0.001), except for the pinch (with the BI score at 3W, 3M and 6M) and grip (with the BI score at 6M) subscale scores with a fair correlation with *ρ* value = 0.440–0.497 (*p* < 0.01). The C-ARAT total and subscale scores at 0D had fair correlation with the BI score at 1Y, with *ρ* value from 0.260 to 0.390 (*p* < 0.05, except for the pinch subscale score, *p* = 0.132). The C-ARAT and subscales at 0D showed good predictive ability on BI scores at 3W and 3M with adjusted *R*^2^ value from 0.111 to 0.290 (*p* < 0.01). The C-ARAT and subscales at 0D was not a significant predictor on BI scores at 6M and 1Y with adjusted *R*^2^ value from 0.005 to 0.082 (*p* > 0.05), except that the grip and gross-movement subscale scores at 0D showed good predictive ability on BI scores at 6M with adjusted *R*^2^ value from 0.071 to 0.109 (*p* < 0.05). The C-ARAT showed the best predictive ability with the BI score at 3W and showed the lowest predictive ability with the BI score at 1Y. Among the total and subscale scores, the pinch subscale showed the lowest predictive ability, and the gross-movement subscale showed the highest predictive ability.

## 4. Discussion

This was the first study to explore the responsiveness, predictive validity and floor and ceiling effects of the C-ARAT in people with a first early cerebral infarction. Our results demonstrate that the C-ARAT had moderate to large responsiveness. The C-ARAT had moderate to good correlation with the BI score at 3W, 3M and 6M and had fair correlation with the BI score at 1Y. C-ARAT was a good predictor of BI score within 3M follow-up but not 6M and 1Y follow-up. The C-ARAT showed a notable floor effect at 0D and 3W follow-up and a notable ceiling effect at 3M, 6M and 1Y follow-up.

### 4.1. Floor and Ceiling Effects

Our results demonstrated that the C-ARAT total score showed a notable floor effect at 0D and 3W follow-up, indicating a poor functional UE in most of the participants at admission. Consistent with our results, Hsueh et al. [5] reported that the ARAT showed notable floor effect in 48 early-stage (onset 24 days) first-onset stroke patients. But different from our results, Dorothy et al. found that the ARAT showed no floor effect in 51 early-stage (onset 9.5 days) stroke patients who presented a moderate degree of UE motor dysfunction [35]. Nijland et al. also reported that the ARAT had no floor effect [21]. The difference may reflect the effects of study inclusion criteria requiring proximal arm movement at the time of enrolment [35].

Our results indicated that the C-ARAT showed notable ceiling effects at the latter three time points (3M, 6M and 1Y follow-up). This may to some extent indicate that the participants achieved considerable recovery of their UE function. Similar to our results, Dorothy et al. reported that the ARAT showed a notable ceiling effect at days 14 and 90 after enrolment [35], and Lin et al. reported that the ARAT showed a notable ceiling effect at 30, 90, and 180 days after stroke [20]. But different from our results, both Hsueh et al. [5] and Nijland et al. [21] reported that the ARAT showed no ceiling effects in their studies. In the study of Hsueh et al. [5], they merely evaluated the participants at admission and at discharge without long-term follow-up. Similarly, the study of Nijland et al. [21] evaluated the 18 participants only once, without long-term follow-up.

### 4.2. Responsiveness

Our results demonstrated that the C-ARAT had moderate responsiveness in detecting changes at 0D–3W and 0D–3M and large responsiveness at 0D–6M and 0D–1Y in this study sample. Similar to the first two sessions of our study, Rabadi et al. reported that the ARAT showed moderate responsiveness (SRM = 0.68) during evaluation of 104 early-stage (onset: 16 ± 9 days) stroke patients who were studied with two measurements at admission and discharge with a mean stay of 34 ± 15 days [23]. Hsieh et al. evaluated 57 chronic stroke individuals (mean onset 12.98 ± 7.62 months) with three outcome measures at pre-treatment and post-treatment and found that the ARAT showed large responsiveness (SRM = 0.95) [7], in agreement with the latter two sessions of our study. However, unlike our results, Wei et al. found that the ARAT showed small responsiveness (SRM = 0.22) during evaluation of 27 chronic stroke patients (mean onset 4.92 ± 0.45 years) evaluated with four measurements before and after interventions [24]. The varying results might be due to the different onset times (28.74 ± 15.32 days in this study vs. 4.92 ± 0.45 years in Wei's study) or to different interventions. Furthermore, another three studies reported the responsiveness of ARAT by the method of effect size *d* during evaluation of early-stage stroke patients, with effect size *d* = 0.49–1.390, indicating small to large responsiveness [5, 20, 35]. The differing results might also be due to different onsets and UE performance of participants. Coupar et al. [36] reviewed 288 studies and found that people with less disability were more likely to have better upper limb recovery after stroke. Kwakkel et al. [37] suggested that the length of time passing without improvement may reflect intrinsic cerebral damage and should be considered to be a predictor of the poor recovery after stroke. In addition, because the C-ARAT showed notable floor and ceiling effects, the responsiveness might be influenced by reference to more severe stroke effects or near-normal UE function. Summarising the above previous studies and ours, we can conclude that the responsiveness is affected by the participants' UE function level and the potential for functional recovery of the upper limbs.

Among the four subscales, the grip subscale, similar to the total score, had moderate responsiveness within 3M follow-up and large responsiveness after 6M follow-up. The gross-movement subscale had the highest responsiveness during the first three time periods. These observations might indicate that participants mainly achieved gross-movement improvement at the early stage and then mainly regained grip function improvement within 1 year follow-up. The pinch subscale had the lowest responsiveness at each session. This might indicate that the pinch function was the most complicated and difficult to recover.

### 4.3. Predictive Ability

Our results showed that the C-ARAT total score showed moderate to good correlation with the BI scores at 3W, 3M and 6M and exhibited fair correlation with the BI score at 1Y after enrolment. In agreement with our results, the study of Lin et al. showed a moderate correlation (*ρ* > 0.5) between the ARAT score at 14 days after stroke and the BI score at 180 days after stroke during evaluation of 53 individuals with early stroke (onset within 2 weeks) [20]. Meanwhile, Hsieh et al. [7] reported that the ARAT had a low predictive validity with the FIM (ρ = 0.17–0.26). They evaluated 57 chronic stroke individuals (onset at least 6 months before) with three outcome measures at pre-treatment and post-treatment. The BI is mainly used to evaluate the independency in terms of mobility and personal care [38]. FIM assesses not only the ability of ADLs but also the ability of community interaction [39]. The low validity may indicate that the components of C-ARAT are not directly associated with the ability of community interaction. In our study, we examined the predictive validity of the C-ARAT total and subscales with the BI score after 1 year, which was a relatively long-term follow-up. In addition, we found that the pinch subscale had the lowest correlation with the BI. This might indicate that, even without fine hand function, an individual may also score high on the BI by performing ADLs with compensatory strategies. In a word, our findings supported the predictive validity of the C-ARAT.

Interestingly, we found that the C-ARAT may be a good predictor of the level of ADL within 3M follow-up. C-ARAT may be an optimal predictor of ADLs in acute and subacute phases of stroke. Kwakkel found that the severity of upper limb motor impairment at onset of the stroke had some impacts on the probability of regaining the upper limb motor function in the acute phase of stroke [40]. In this study, the result may reveal that the improvement in ADLs can be attributed mainly to the improvement of upper limb motor function in acute to subacute phases of stroke. Kwakkel's review also suggested that the functional recovery plateaus occurred 3–6 months after stroke on average [41]. The improvement in ADLs was determined not only by the upper limb motor function but also by some other rehabilitation factors, such as the functional compensation strategy [42], psychological factors [43], rehabilitation aid equipment and environment adaptation [44]. This may indicate that the C-ARAT is an especially good tool to predict the ADL level in acute and subacute phases of stroke. It may show potential clinical value in prognosis and decision-making in treatment schemes for acute stroke rehabilitation.

This study had some limitations. First, the study subjects were selected by convenience sample method. The subjects were recruited from the hospital in their acute phrase of stroke. Most of them showed a strong will towards stroke rehabilitation. This attribute may have led to the over-representation within the sample of particularly active individuals. Second, the sample size was small at the follow-up of 1Y. The conclusion may only apply to the subjects in our study. A larger sample size with different phases of stroke may help us to draw a more generalisable conclusion. Third, enrolment onset was 4–81 days, which was a somewhat large range and might somehow have some impacts on the results. Our study found that C-ARAT can predict BI from acute to subacute stroke. Although all the subjects were recruited in acute phase of cerebral infarction, the registration time after cerebral infarction was various. It may somehow deteriorate the result in our study. In order to minimize the variation in the onset and draw a more reliable conclusion, subjects within 2 weeks post stroke [20] would be warranted in the further study. Fourth, we only evaluated the responsiveness, predictive validity, and floor and ceiling effects of the C-ARAT in this sample. To gain a deeper understanding of C-ARAT, further research should be carried out to explore the comprehensive psychometric characteristics.

## 5. Conclusion

The C-ARAT showed acceptable levels of responsiveness in people with cerebral infarction. C-ARAT was a good predictor of ADL performance in acute and subacute phases of cerebral infarction. Our results support the use of the C-ARAT as a valid measure of UE impairment in individuals with a first cerebral infarction.

## Figures and Tables

**Table 1:** Characteristics of the study participants.

Variable	0D (97)	3W (91)	3M (59)	6M (42)	1Y (35)	Withdraw (62)	Complete (35)	*P*
	Values	Values	Values	Values	Values	Values	Values	
Age (years)	59.87 ± 10.94	59.95 ± 10.79	60.10 ± 10.61	58.69 ± 11.71	58.34 ± 11.06	60.73 ± 10.87	58.34 ± 11.06	0.306
Onset (days)	28.74 ± 15.32	50.67 ± 15.62	118.02 ± 16.37	211.76 ± 19.78	397.26 ± 20.39	28.94 ± 15.30	28.40 ± 15.58	0.870
Sex								
Male	75 (77.3)	71 (78.0)	47 (79.7)	33 (78.6)	26 (74.3)	49 (79.0)	26 (74.3)	0.592
Female	22 (22.7)	20 (22.0)	12 (20.3)	9 (21.4)	9 (25.7)	13 (21.0)	9 (25.7)	
Affected side								
Right	52 (53.6)	50 (54.9)	31 (52.5)	20 (47.6)	15 (42.9)	37 (59.7)	15 (42.9)	0.111
Left	45 (46.4)	41 (45.1)	28 (47.5)	22 (52.4)	20 (57.1)	25 (40.3)	20 (57.1)	
BI	53.04 ± 24.93	65.33 ± 25.08	74.75 ± 23.75	85.95 ± 17.50	87.29 ± 16.69	49.60 ± 24.47	59.14 ± 24.93	0.070
C-ARAT total	14.55 ± 18.67	18.09 ± 20.39	21.81 ± 22.12	27.98 ± 22.89	28.77 ± 23.32	13.42 ± 17.28	16.54 ± 21.02	0.432
Grasp score	4.55 ± 6.43	5.58 ± 6.96	6.88 ± 7.48	9.12 ± 7.86	9.23 ± 7.76	4.21 ± 6.09	5.14 ± 7.03	0.495
Grip score	3.02 ± 4.11	3.81 ± 4.53	4.73 ± 4.92	6.02 ± 5.02	6.23 ± 5.20	2.82 ± 3.90	3.37 ± 4.49	0.530
Pinch score	3.36 ± 5.90	4.42 ± 6.55	5.32 ± 7.44	7.38 ± 7.79	7.66 ± 8.04	2.94 ± 5.36	4.11 ± 6.77	0.380
Gross movement score	3.62 ± 2.93	4.27 ± 3.05	4.88 ± 2.97	5.45 ± 2.94	5.66 ± 3.10	3.45 ± 2.73	3.91 ± 3.27	0.458

Note. Values are mean ± SD or *n* (%). *P* < 0.05 indicates significant correlations. Abbreviations: C-ARAT, Chinese-version of Action Research Arm Test; BI, The Barthel Activities of Daily Index; D, Day; W, Weeks; M, Months; Y, Year.

**Table 2:** Floor and ceiling effects of the C-ARAT at 0D, 3W, 3M, 6M and 1Y follow-up.

	Floor Effect					Ceiling Effect				
	0D (97)	3W (91)	3M (59)	6M (42)	1Y (35)	0D (97)	3W (91)	3M (59)	6M (42)	1Y (35)
C-ARAT	28 (28.9)	20 (22.0)	7 (11.9)	4 (9.5)	4 (11.4)	8 (8.2)	9 (9.9)	13 (22.0)	10 (23.8)	10 (28.6)
Grasp	58 (59.8)	48 (52.7)	27 (45.8)	16 (38.1)	13 (37.1)	8 (8.2)	12 (13.2)	12 (20.3)	11 (26.2)	10 (28.6)
Grip	57 (58.8)	46 (50.5)	26 (44.1)	14 (33.3)	11 (31.4)	3 (3.1)	7 (7.7)	11 (18.6)	11 (26.2)	11 (31.4)
Pinch	66 (68.0)	56 (61.5)	32 (54.2)	16 (38.1)	15 (42.9)	5 (5.2)	9 (9.9)	11 (18.6)	10 (23.8)	11 (31.4)
Gross movement	28 (28.9)	20 (22.0)	7 (11.9)	4 (9.5)	4 (11.4)	10 (10.3)	15 (16.5)	15 (25.4)	12 (28.6)	13 (37.1)

Note. Values are *n* (%).

Abbreviations: C-ARAT, Chinese-version of Action Research Arm Test; D, Day; W, Weeks; M, Months; Y, Year.

**Table 3:** Responsiveness of the C-ARAT at different recovery stages.

Days after recruit (No. of participants) and scale		Change score	SRM	Wilcoxon Test	
		Mean ± SD		*z*-value	*p*-value
0D-3W (91)	C-ARAT	4.11 ± 7.03	0.58	−5.753	<0.001
	Grasp	1.23 ± 2.70	0.46	−4.160	<0.001
	Grip	0.91 ± 1.72	0.53	−4.814	<0.001
	Pinch	1.21 ± 2.74	0.44	−4.020	<0.001
	Gross movement	0.76 ± 1.32	0.58	−4.741	<0.001

0D-3M(59)	C-ARAT	7.75 ± 10.75	0.72	−5.240	<0.001
	Grasp	2.59 ± 4.07	0.64	−4.067	<0.001
	Grip	1.95 ± 2.67	0.73	−4.554	<0.001
	Pinch	1.88 ± 4.13	0.46	−3.735	<0.001
	Gross movement	1.32 ± 1.72	0.77	−4.670	<0.001

0D-6M (42)	C-ARAT	11.48 ± 14.19	0.81	−4.953	<0.001
	Grasp	3.95 ± 5.39	0.73	−3.886	<0.001
	Grip	2.60 ± 3.25	0.80	−4.205	<0.001
	Pinch	3.38 ± 5.24	0.65	−3.935	<0.001
	Gross movement	1.55 ± 1.88	0.82	−4.231	<0.001

0D-1Y(35)	C-ARAT	12.23 ± 14.49	0.84	−4.787	<0.001
	Grasp	4.09 ± 5.36	0.76	−3.539	<0.001
	Grip	2.86 ± 3.19	0.90	−4.119	<0.001
	Pinch	3.54 ± 5.59	0.63	−3.521	<0.001
	Gross movement	1.74 ± 2.21	0.79	−4.009	<0.001

Note. *P* < 0.05 indicates significant correlations. Abbreviations: C-ARAT, Chinese-version of Action Research Arm Test; SRM, Standardised Response Mean; D, Day; W, Weeks; M, Months; Y, Year.

**Table 4:** Predictive ability of the C-ARAT at 0D on BI scores at 3 W, 3 M, 6M and 1Y follow-up.

	BI score (3W, *n* = 91)				BI score (3M, *n* = 59)				BI score (6M, *n* = 42)				BI score (1Y, *n* = 35)			
	Spearman's		Regression		Spearman's		Regression		Spearman's		Regression		Spearman's		Regression	
	*ρ*	*p*	Adjusted *R*^2^	*p*	*ρ*	*p*	Adjusted *R*^2^	*p*	*ρ*	*p*	Adjusted *R*^2^	*p*	*ρ*	*p*	Adjusted *R*^2^	*p*
C-ARAT (0D)	0.581	<0.001	0.249	<0.001	0.624	<0.001	0.185	<0.001	0.561	<0.001	0.054	0.075	0.384	0.023	0.039	0.133
Grasp (0D)	0.550	<0.001	0.232	<0.001	0.603	<0.001	0.188	<0.001	0.521	<0.001	0.057	0.069	0.391	0.020	0.042	0.124
Grip (0D)	0.571	<0.001	0.254	<0.001	0.614	<0.001	0.202	<0.001	0.497	0.001	0.071	0.049	0.360	0.034	0.051	0.102
Pinch (0D)	0.451	<0.001	0.187	<0.001	0.471	<0.001	0.111	0.006	0.440	0.004	0.011	0.235	0.260	0.132	0.005	0.290
Gross (0D)	0.579	<0.001	0.290	<0.001	0.614	<0.001	0.261	<0.001	0.532	<0.001	0.109	0.019	0.383	0.023	0.082	0.053

Note. The *ρ* values indicate correlation coefficients by Spearman's rank correlation coefficient. The adjusted *R*^2^ values indicate coefficients by linear regression. *p* < 0.05 indicates significant correlations. Abbreviations: C-ARAT, Chinese-version of Action Research Arm Test; BI, The Barthel Activities of Daily Index; D, Day; M, Months; Y, Year.

## Data Availability

The data used to support the findings of this study are available from the corresponding author upon request.
